# "The Hospital" Nursing Mirror

**Published:** 1899-11-04

**Authors:** 


"The Hospital" Nursing Mirror.

Being the Nursing Section of "The Hospital."

[Contributions for this Section of "The Hospital" should be addressed to the Editor, The Hospital, 28 & 29, Southampton Street, Strand, London, W.C., and should have the word "Nursing" plainly written in left-hand top corner of the envelope.]

### Notes on News from the Nursing World.

### NURSES AND THE WAR.

It is stated in one of the daily papers that "should the situation demand that 5,000 nurses be sent to South Africa that number of efficient ladies could be procured at a day's notice, so inspired are member of the profession by the teachings of Florence Nightingale, and with the example of many other 'ministering angels,' who have served their country under fire as well as Tommy Atkins himself." Five thousand is a big number of efficient nurses to procure "at a day's notice," and happily they are not likely to be required. But there is not the slightest fear of any demand, however great or urgent, not being met. The volunteers for the front are very far in excess of those needed.

### THE DEPARTURES FOR SOUTH AFRICA.

On Saturday the first contingent of Army Nursing Reserve left Waterloo Station in order to join the "Carisbrook Castle" at Southampton, namely, Miss Falcon, Miss Sainsbury, Miss Watson, Miss Babb, and Miss Woollcomb. The blue cloaks and red hoods made a conspicuous, though neat and becoming costume amongst dresses which ranged from the height of eccentric fashion to the simplest of coats and skirts. In the same train were the following members of the Army Nursing Service: Sisters Hardement (superintendent), Macintyre, de Montmorency, Barker, Maclean, Beck, Kinahan, and Culverwell. Prior to the departure of the "Carisbrook Castle," Colonel Young (the organiser of the Red Cross Society) who accompanied the nurses, was received by the Princess of Wales at Marlborough House. The next departure will include Sisters Browne, Wilson, Cox, Potter, Palmer, Rose, Harding, and Steen, and Nurse Whiteman of the Reserve. Miss Billjohns, Miss Noble, and Miss Alice Brown are also ordered to proceed to South Africa.

### PERSONAL NOTES ABOUT THE WAR NURSES.

Miss Hardement, who has gone out as superintendent, was trained at Guy's Hospital, and has been a member of the Army Nursing Service for several years. Miss M. C. Rose has been for some time sister at the Guards' Rochester Row Hospital, where she will be replaced by Miss A. G. Wason of the Army Nursing Reserve. Miss Browne, of West Bromwich, a former matron of the district hospital, was in the Soudan campaign. Miss Noble is matron of the Beverley Cottage Hospital, and a member of the Princess Christian Nursing Association. Miss Alice Brown, who is also a member of the association, has been up to now sister in charge of the typhoid wards at the Hull Corporation Sanatorium. Miss Billjohns, who goes to Ladysmith, has seen service in the Chitral, Afridi, and Omdurman campaigns, and is the daughter of a general.

### NURSES AT NATAL.

Before Parliament was prorogued the Under-Secretary for War stated that there were five nursing sisters in Natal. By this time there are 1,100 hospital beds at the disposal of the authorities. In addition to this, there is at Wynberg, near Cape Town, a hospital with 520 beds, and the "Spartan" and the "Trojan" have been sent out to make that institution available as a base hospital for the force in Natal. The first of these transports, which, as Mr. Wyndham says, are specially fitted up for the conveyance of invalids between Durban and Cape Town, should reach Durban this week, and the other ten days later.

### THE WOMEN AND CHILDREN WE LEFT BEHIND US.

A medical man in South-west London, who only refrains from giving his name that there may be no appearance of advertising himself, declares his readiness, and that of his assistants and nurses, to gratuitously attend the women and children of soldiers "married without leave," and of Army Reservists who may now be sick. So far as nurses are concerned, we are quite sure that there are many members of the profession willing to render what help they can in this way. Those who cannot afford to subscribe to any fund for the benefit of the sick and wounded can, by means of personal service, do much to ameliorate the hard lot of the poor creatures whose bread-winners are risking their lives for their country; and who, in their absence, will find the burden of illness exceptionally grievous to be borne.

### THE REPORTED EVICTION OF ENGLISH NURSES AT JOHANNESBURG.

Last week mention was made in our pages by a correspondent from Durban of a Boer farmer near Harrismith who, before the war broke out, said that "if he were wounded and an English nurse offered him brandy, woman or no woman, he would shoot her if he could." Almost on a parallel with this is the reported eviction of English nurses from a hospital at Johannesburg, on the ground that "no Englishwoman is fit to nurse a Dutchman." Such, at least is said to be the dictum of Dr. Mangoldt. But as there appears to be no medical man of that name attached to the Johannesburg Hospital, which was founded in 1888, and has a large nursing staff, it is possible that the report may have reference to a minor institution, like the Jumpers' Hospital.

### MISS FLORENCE NIGHTINGALE ON "THE ADVANTAGES OF WAR."

By far the most interesting incident at the dinner given to the survivors of the Balaclava Charge was the reading of a letter from Miss Florence Nightingale. Although Miss Nightingale truly says that few men, and perhaps no women, have seen as much of the horrors of war as she has, yet she urges that there are advantages of war, as well as of persecution. To quote her own graphic language: "But see those manly fellows in time of war, men not near the beasts, as sometimes we too sadly see in the time of peace; see them not one taking a drop too much; not one gallivanting with the women; every one devoting, aye, even his life for his comrade, fetching his comrade off the field, without notice or praise from anyone, either in words or in print; and if killed in the attempt, his name only goes down as 'killed in battle'; always devoted even to the death, as our Great Master and Friend, Jesus Christ, was to His fellow men." Since the letter was written, a correspondent of a Liverpool paper states that Miss Nightingale was consulted by the military authorities with regard to the hospital arrangements in the Transvaal. It must be gratifying to her to find that, even in the changed conditions of military nursing, her advice is still valued at the War Office.

### THE NURSES AT WIRRAL HOSPITAL.

In the spring there is to be a big bazaar in Birkenhead, in aid of the Children's Wirral Hospital. "Some years ago," says a correspondent, "I nursed a private case at a house, from the bedroom of which I could see distinctly into one of the long wards. I often saw the nurses hug and kiss the children, and sometimes one nurse would run about the ward with a child in her arms, another pretending to chase them, all the little ones in the cots enjoying the fun immensely. Occasionally a nurse left the ward for a minute, and at once down went all the bed-clothes, to be hastily scrambled back as the ward door opened. Then the poor nurse went patiently round straightening them all up again. If it happens to be either of those two who was the nurse who went to South Africa lately, Tommy Atkins is in luck."

### THE WORK OF THE ST. JOHN AMBULANCE ASSOCIATION.

It is gratifying to learn that the response to the appeal of the St. John Ambulance Association is even in excess of expectations. Whatever may be the difficulties of nurses in tending the sick and wounded soldiers, there will be no lack of suitable articles of clothing for the sufferers. Money and gifts in kind have poured in to the offices of the association during the last few days. For the information of those who are sending the latter, we may state that gifts for the first shipment should be sent to the association's warehouse, care of Messrs. Barnes and Co., Ltd., Battlebridge Lane, Tooley Street, E.C., and should arrive there not later than the 14th instant.

### A FEATURE OF NURSING AT WARRINGTON.

In the second annual report, just issued, of the Warrington District Nursing Association, it is observed that "the number of housewives nursed is very striking." Out of a total of 291 patients 155 were housewives. "In many cases," says the report, "the poor women are left to the care of children much too young to understand the importance of anything but play, and their gratitude for the ministrations of the nurses, whereby they are made clean and comfortable, and are fed, at least once a day, often finds expression in the cry, 'I'd have been lost but for you, nurse.'" The nurses' home has been visited by an inspector from head-quarters, and her report is most satisfactory.

### WORKHOUSE NURSES AND THE BATHING OF PATIENTS.

A correspondent writes: "At a board meeting of the Tendring Guardians recently, the master of the workhouse reported that a newly-appointed nurse had refused to bath a male patient, or to be present in the bath-room during the time that male patients were being bathed. The master told the nurse she must either do it or be dismissed from duty. She chose the latter course, and left almost immediately. The clerk read a letter to the board in which Nurse Norton appealed to them on behalf of her instant dismissal by the master. She added that she refused to bath the male patients on the grounds that she thought it indecent that a female nurse should have such duties to perform, and she asked for the Guardians' further consideration of the matter. A motion was moved and carried approving the master's action in the matter. Considering that most patients in small union infirmaries are chronic, and well able to be bathed by men, it can scarcely be necessary that a nurse should do more for such cases than prepare and test the bath, see the patient to and from the bath, and remain within hearing during the time he is being bathed. The helpless and painfully crippled patients, as also all acute cases, are blanket-bathed by a nurse; but is there any reason why a strong convalescent or an able-bodied inmate should not perform this very important and necessary function for other patients under a nurse's supervision?"

### INFIRMARY NURSES AND FEVER PATIENTS.

Certain individuals in Paisley having accused the infirmary nurses of ill-treating their fever patients, an ex-patient who was for six weeks an inmate of the fever ward comes forward with a vigorous protest. Speaking with the weight which only attaches to personal experience, he says, "I never saw any ill-treatment, and I can testify that we received much kindness and attention from all the nurses, both day and night. Of course, there are some patients who are difficult to deal with and give the nurses more trouble than others, and there are sometimes little ones who will not take their medicine and have to be forced to do so for their good." "But," he adds, "the nurses do their best to keep everybody cheery, and it makes me angry when I hear people saying hard things about them." We have no doubt that this is the ordinary experience of fever patients south, as well as north, of the Tweed.

### A NURSE'S BICYCLE.

Nurses who cycle cannot always obtain accommodation for the storage of their machines when they are not using them. Some of them may like to hear of a bicycle designed by Nurse E. C. Norris, of Bath, called the "Compactum." It is to be exhibited at the National Cycle Show, which opens on the 17th at the Crystal Palace, and the peculiarity is that the handle-bar is readily detachable. When it is disconnected the bicycle, it is said, takes up very much less space than ordinarily. Another advantage claimed for the machine is that by taking off a part of the handle-bar it is rendered difficult to ride, and therefore not likely to be stolen.

### THE NURSES' HOSTEL.

The report of the Nurses' Hostel Company, which was adopted at the annual meeting last week, is of a satisfactory character. During the past year the accommodation of the hostel has, it is stated, "frequently been taxed to the uttermost, and the management has at times been obliged to refuse guests." This is reasonably regarded as a proof that the hostel meets a want in the profession. The income for the year was £2,111, and the expenditure £1,638, leaving a balance of £473. A dividend of 3½ per cent. has been declared, and a reserve fund has been started with a sum of £100. Miss Wood and Sir Allen Johnson have been re-elected directors.

### NURSING IN SCOTCH WORKHOUSE INFIRMARIES.

Both the workhouse infirmaries in Edinburgh are practically in the country. Craiglockart is attached to the poorhouse, but Craigleith is quite a separate building. "The work for the nurses," writes a nurse from Edinburgh, "is hard, because they have about 40 patients each to look after, and at Craigleith there are so many helpless old men and women. The nurses appear to be bright and happy notwithstanding, and the salary is from £28 a year upwards. The superintendents of nurses of both hospitals know exactly what work can be done, as they were nurses in the infirmary before they became superintendents. The Parish Council are the House Committee. There are two ladies on the Poorhouse Committee, and they have done wonders for the poorhouse infirmary. Craigleith is really splendidly managed and beautifully arranged. The ward floors are all polished wood, linoleum is laid down the centre of the wards, the fireplaces are also in the centre, and each bed has a mat 3 feet by 2 feet. They look very comfortable. The nurses get off one half-day a week, I believe. There are seven nurses at each poorhouse infirmary; two are always on night duty, leaving five for day duty. The maternity wards are quite separated from the other part of the infirmary. The nurses' home is very comfortable, and their bedrooms are very fair sized rooms, very airy, and could be made very pretty if the nurses chose to make them so. Being so far from the town three of the nurses have their own bicycles. Going round with one of the ladies of the Parish Council I could not help thinking how very comfortable and happy they all seemed. The Hospital is always taken in, and is looked forward to every week."

### THE JESSOP HOSPITAL FOR WOMEN, SHEFFIELD.

In the annual report of the Jessop Hospital for Women prominence is given to the fact that the extension, for which subscriptions are now being solicited, will include new nurses' quarters and the better lodgment of the maternity department. It is estimated that the cost of the extension will be about £20,000, of which sum a considerable proportion has been promised. The hospital was opened 35 years ago with six beds, and was transferred to its present position in 1878, the site, buildings, and complete furnishing being paid for by the late Mr. Thomas Jessop, president of the hospital, who also left £4,000 to it in his will. Other members of the Jessop family have liberally assisted it, and no doubt the Sheffield public will find the balance still required.

### DWELLINGS FOR LADIES.

Nurses will learn with interest that the Women's Industrial Council is undertaking an inquiry into the wants and desires, in the matter of housing, of ladies living and working in London. The great need of suitable accommodation for educated women is at last forcing itself upon public attention, and many schemes are taking shape. It is felt, however, that very little is known as to the real requirements of the women personally concerned, and that they themselves can best supply this information. A form of inquiry has been drawn up, and is being extensively circulated. The returns will be carefully tabulated, and a report issued. We understand that the answers already received show that the inquiry is heartily welcomed, that there is great readiness to reply to it, and that there is a crying need for something further or different in the way of accommodation. Ladies desiring forms to fill up, or for distribution to others for filling up, can obtain them from the office of the council, 12, Buckingham Street, Strand, W.C.

### THE INTERNATIONAL HOSPITAL, NAPLES.

The International Hospital at Naples is called after one of its benefactresses, Villa Bentinck. It is not free, but of course extremely necessitous and dangerous cases are always received. The chief idea about it is to be international, and it is principally for sailors, &c., stopping in the port. The patients are divided into classes, according to whether they pay 15 or 2.50 lire a day; 191 cases were admitted last year, and of these 58 were English, chiefly sailors. There is one bed specially reserved for English patients, called the Victoria Bed; it is a souvenir of the reign of the Queen. There is also a British Relief Fund, which contributes towards the expense of a bed. The British Consul takes an interest in the hospital, and British patients are always particularly well cared for. Miss Moore is matron and superintendent, and her services are very highly valued. She personally superintends the training of the nurses who are English and Italian, and are obliged to speak German. All the requirements are as modern as possible.

### SHORT ITEMS.

We are glad to hear that Miss Gordon, the matron of St. Thomas's Hospital, is quite convalescent from her recent illness, and expects to resume her duties this month.—Nurses Coulson, Riddick, and Poole have arrived in Bombay. They are the first nurses sent from St. Thomas's Hospital for plague duty.—An association affiliated to the Jubilee Nurses' Institute has been formed in Killarney for the benefit of the poor of the town and district.—The "Braemar Castle," containing Miss Garriock, of the Herbert Hospital, Woolwich (superintendent), and Sisters Rose-Innes, Neale, Anderson, Nixon, Murphy, Snowden, and Guthrie, has arrived at Cape Town.—President Steyn has accepted the offer of the Sisterhood of St. Michael at Bloemfontein to nurse the wounded Boers.—Letters sent to nurses tending the sick and wounded should be addressed to them by name, "Army Nursing Service, Cape Town, South Africa. To be forwarded."—Under the will of Miss Anne Elizabeth Rolland, of Holmwood, Weybridge, the sum of £300 is bequeathed to the Metropolitan and National Nursing Association.—A nurses' home has been opened by the Earl and Countess of Moray in connection with the Northern Infirmary, Inverness. —At a recent meeting of the Executive Committee of the Royal British Nurses' Association, Miss de Pledge was unanimously appointed to represent the Association on the council of the National Union of Women Workers. Princess Christian nominated Miss de Pledge from the chair, and the proposal was supported by Mr. Langton and Mrs. Coster.—The nurses of the Norfolk and Norwich Hospital have learnt, with deep regret, of the resignation of their matron, Miss Adam, who for 23 years has been to them all a true friend and trusted adviser.

### Lectures to Surgical Nurses.

By H. A. LATIMER, M.D. (Dunelm), M.R.C.S. of Eng., L.S.A. of London, Consulting Surgeon, Swansea Hospital; Past President of the Swansea Medical Society; Lecturer and Examiner of the St. John Ambulance Association, &c.

### ANEURISM—DESCRIPTION OF THE DISEASE AND EFFECTS PRODUCED BY IT—ITS CAUSES.

TO-DAY I propose to lecture to you on the subject of aneurism, that formidable disease of the arteries which, when it occurs in blood vessels within the body, comes under the care of the physician, and when it shows itself in the limbs and external parts is treated by the surgeon. As this affection of the blood-vessels is responsible for much suffering and for many deaths, it is of great importance that it should be recognised at an early stage of its appearance, for it is then that a greater measure of success attends our efforts to cure it than at a later time, when it has become thoroughly established.

Let me first of all tell you what constitutes an aneurism; having done this, I will instruct you how it forms, what it gives rise to, and how surgeons proceed to cure it.

An aneurism is a tumour formed by the yielding of one or more coats of an artery. I should tell you that the arteries are made up of three coats—an inner, a middle, and an outer one. I have told you in a previous lecture what the function of these vessels is—that they are channels for the transit of blood from the heart to all parts of the body. I have also told you that they have elastic and muscular coats, and that they dilate to admit of the successive waves of blood as these are sent by energetic contractions of the heart, and that, by their subsequent contraction, they pass this blood onwards in the direction in which it should travel.

Bearing these physiological details in mind, you will readily appreciate tho fact that unless the arterial walls are in a proper state of health, as they are constantly being subjected to great strain, some mischief will easily happen in them. They can never be at rest unless the circulation through them is arrested by disease or by artificial interference, and if this passage of blood is stopped the gravest results are apt to follow in the organs they convey blood to, because with the deprival of this blood must come a loss of nourishment to those parts, unless the burden of supplying the latter with this vital fluid can be taken up by new channels.

I will proceed by describing an aneurism as it shows itself in a part which is accessible to sight and touch. Such a part is to be found in the ham—the space at the back of the knee—where these tumours often occur by dilations of the popliteal arteries. In a case such as I am speaking, of a throbbing, pulsating swelling will appear; it will manifest itself both to sight and to touch, and it will be found on examining it by the latter sense that as it pulsates it not only possesses a heaving impulse but that it also has a distending beat, and that it pushes apart from each other two fingers which have been placed on opposite sides of it. When this last fact has been proved it is certain that we are dealing with an aneurism, for it shows in an unmistakable manner that the tumour which is being examined is inherently expansile, and that its movements are not communicated to it from adjacent parts.

The key to the knowledge of what has taken place in the diseased artery under review is afforded by a study of the changes wrought in one or more of its walls by processes of inflammation or degeneration. Here, as elsewhere in the body, the result of an inflammation is the pouring out into the parts of the organ concerned, from the fine blood vessels which nourish them, of a number of small cells which subsequently undergo reabsorption, or change into fibrous structures, or alter the parts in which they lie into fatty or lime-like substances. If the walls alter at all their integrity is destroyed, and they yield to the pressure of the waves of blood, which distend them in regular sequence; and when they have so yielded a more or less diffused swelling results, and we have an aneurism. Here is the matter in a nutshell. There are a number of refinements of the subject and classification of the tumours themselves, according to their shapes, the numbers of the coats involved, and whether these coats have only yielded to pressure, or whether they have broken and so allowed of the escape of blood itself. With such refinements you are not concerned; for you it is sufficient to know of their existence.

I think it will be interesting if I tell you why the coats of the arteries give way when inflammation has occurred in them, so I will devote a few minutes to this part of my subject. The great reason for their doing so is to be found in the anatomical fact that the nourishing vessels to the arterial walls are distributed only to the two outer ones, and that the inner coat has no direct supply of its own. As a consequence of this peculiarity when the parts are swollen by the presence in them of the round cells which are a product of the inflammatory process, they undergo a degeneration, and here and there change into fatty and pasty patches. Any one of these fatty patches may soften down into a minute abscess, and if this abscess behaves just like an abscess does elsewhere, it will discharge into the blood stream, and then there results a weak spot in the wall of the artery by which blood can get in between its coats and so dissect them up and cause them to yield to pressure. But in the same vessel we may have other patches which have formed plates of lime salts degeneration, and as these differently weakened spots yield to blood pressure we have irregular dilatations. And all these dilatations constitute aneurism.

We will next concern ourselves with the causes of this condition of the vessels. Foremost in this respect are syphilis and the abuse of alcohol, and great physical and mental exertion also decidedly lead to it. The combination of drink with strong and long-sustained bodily effort very notably leads to degenerations in the walls of the blood vessels.

When once set up the disease is pretty sure to progress, and the symptoms induced by it are of the most distressing character. For it is a peculiarity of aneurisms that, although they are formed of soft materials, they have the power of stretching parts in their neighbourhood, and of even eating away gristle and bone which may be in their way as they enlarge under the constantly-increasing distension to which they are subjected. In this way they set up the so-called "pressure symptoms" which you will hear surgeons discussing as they stand by the bedsides of patients who are suffering from this formidable disease—"pressure symptoms" being nothing more nor less than the effects produced upon arteries, veins, nerves, bones, and internal organs, which are interfered with, pushed aside, eroded, or blocked up by the intruding blood tumour. I will give a few instances of the result of pressure upon different parts as an illustration of the subject.

If the aneurism should occur within the chest, at one part of the great artery which lies near the heart, it is apt to press upon and to obliterate the great vein which brings blood back from the head and neck and arms. As a consequence of such interference with returning blood the parts which should be drained of it remain soddened, and dropsy declares itself there. At the same time, nerves being pressed upon and bronchial tubes being also obstructed, neuralgias set in, and troublesome cough and difficulty of breathing are also set up.

Take an instance of "pressure symptoms" on bone. An aneurism of the great artery in the thorax will sometimes make its way to the skin of the back, and in so doing will actually perforate obstructing ribs, nothing being able to stand in its way. You would naturally think that it would make for the apparent point of least resistance, and that it would inevitably push towards the soft organs enclosed within the chest. But no; should the dilating part of the artery present itself towards the hard enclosure of ribs, these have to give way and undergo absorption in response to the constant encroachment upon them of the pushing intruder. I shall give you one or more instances of this law when I resume the subject in my next lecture.

### The Colonial Nursing Association.

### I.—ITS ORIGIN.

The world owes many of the most admirable movements which have been set on foot for the benefit of the human race to women. As even people with short memories know, it was a woman who was instrumental in effecting a revolution in nursing, and it is in accordance with the fitness of things that where Florence Nightingale led others of her sex should follow. The Colonial Nursing Association, in reference to which Mr. Chamberlain said at the last annual meeting, "The Colonial Office are willing, to the very utmost of their power, to give the co-operation which is needed, and in every way to support the work," owes its existence to the womanly solicitude and resolution of Mrs. Francis Piggott. But for her thoughtfulness in conceiving the scheme, her skill in carrying it into effect, and her unwearied exertions in obtaining support both at home and across the seas, there would have been no Colonial Nursing Association. She is one of the last persons to desire that her fame should be noised abroad, but "honour to whom honour is due," and it is only fair and proper that the name of Mabel Piggott should always be inseparably associated with a movement which, there is now every reason to believe, is destined to grow from a small beginning into a potent agency for the amelioration of suffering English people in distant parts of the Empire.

From the outset Mrs. Piggott has received nothing but kindness from Mr. Chamberlain, and his co-operation has been, of course, most valuable. But in no sense is the enterprise that of the Colonial Office. For half a century the Colonial Authorities were, perhaps, unavoidably inactive, and neither by them, nor by anyone else, was a project for the solution of the nursing problem in the Crown colonies propounded, until Mrs. Piggott put her hand to the plough, and became the pioneer of a work which she has carried to a successful issue. It was not, indeed, until the Colonial Office realised that she had proved it could be made a success, that they decided to take it up. Perhaps the argument of the department would be that they could not identify themselves with a possible failure, and it must be added that their support, once given, has not been at all half-hearted.

But how was it that Mrs. Piggott got the idea of the Colonial Nursing Association into her mind? Her husband, Mr. F. T. Piggott, is Procureur and Advocate-General in Mauritius, and it was while she was residing in the island that she was painfully impressed with the utter lack of nursing afforded so far as the upper classes were concerned. It is true that there is a Colonial Government Hospital in the island for the use of the natives and sick poor, with a staff of willing if quite untrained French sisters; but in 1895 there was not a single nurse available for other persons, from the Governor's household downwards. This fact was brought home to Mrs. Piggott by the deaths of three young Englishmen from typhoid on one sugar estate alone, and by the knowledge that in each instance death resulted from utter neglect and the absence of any kind of nursing save that afforded by ignorant native boys. It was accentuated by a most distressing incident. The wife of an English officer in the Black Watch, who was expecting her confinement, had her baby of fifteen months old at death's door, the victim of malaria and dysentery. The only person to attend upon them was a native woman, known, without any intention of irony, as *une femme sage*. As may be imagined, the young parents were nearly distracted; and it was while nursing their child, day and night for a fortnight, that Mrs. Piggott was aroused to the keenest indignation that, in a colony quite able to support a nurse, there was not one to be obtained on any conditions whatever.

Her concern was not, however, for Mauritius only. She saw that the situation must be the same elsewhere, and, though her personal experiences finally determined her to move in the matter of reform, she decided to frame a scheme that would apply to all the Crown colonies. The basis of it was the formation of a central organisation in England, to which the colonies might apply for help in the selection and dispatch of nurses for work abroad, as well as for financial aid when required; and the first move taken to put it into execution was the submission of the plan to several colonial Governors and medical officers. In each case a favourable reply was received, and, encouraged by these promising communications, the founder proceeded on her course. And here, it may be explained, in reference to the question of financial aid, that though Mauritius did not require much assistance, Mrs. Piggott ascertained that many other colonies were not in the same happy position. While a proportion of them were willing to start a nurse and defray the cost of her maintenance and salary, they could not afford to pay her passage out; others could do nothing except provide the nurse with free quarters in a Government hospital. Each of these cases had to be met, and from the commencement the principle of helping those who were willing to help themselves as best they could, was steadily kept in view. In some instances the only help required was to secure the nurse, whom they were willing and able to pay.

At the end of 1895 Mrs. Piggott arrived in England, armed with letters of approval from governors, medical officers, and others in the colonies, and at once began to try and interest people at home in her proposals to rescue our fellow countrymen from the danger of sickness in tropical lands without the presence of a trained nurse—a danger which she was convinced needed merely to be pointed out in order to be removed. Strangely enough, she found that the persons most willing to support the scheme here were, not those who had relations in the Crown colonies, or those who had property in them, and ought therefore to be most ready to come to the rescue, but those who had no personal interest to serve and yet realise that they have a duty in the matter. The small amount which she raised with the object of making an immediate beginning was chiefly subscribed by her private friends, though two or three charitable personages to whom she appealed also contributed, and before the spring had terminated, two nurses had been despatched to Mauritius. Their passage out was paid by the association, then in embryo, and their salaries of £60 each per annum were guaranteed. As soon as the sinews of war were forthcoming it became essential to form a committee to administer the fund, and Mrs. Piggott had the good fortune to enlist the co-operation of Lord Loch, who consented to act as President, and who, together with Mrs. Chamberlain and Lady Knutsford, has been a tower of strength to the movement. The next step was to apply for official recognition from the Colonial Office. It was not supposed that pecuniary help could be given, but Mr. Chamberlain was asked by Mrs. Piggott to recommend the scheme. He consented, and accordingly, in July, 1896, a circular dispatch to all the Governors of the Crown colonies, enclosing the papers of the Colonel Nursing Association, and suggesting their favourable consideration, was sent out under the seal of the Secretary of State. Thus, with the official blessing, came formally into existence the gracious undertaking which had its origin in a ministry of love.

### To Nurses.

In order to increase and vary the interest in the ***Mirror***, **we invite contributions from any of our readers in the form of either an article, a paragraph, or information, and will pay a minimum of 5s. for each contribution. All rejected manuscripts are returned in due course, and all payments for manuscripts used are made at the beginning of each quarter, *i.e.*, January 1st, April 1st, July 1st, and October 1st.**

### The American Hospital Ship.

A CHAT WITH THE HON. SECRETARY.

Although the scheme for starting an American hospital ship to aid the wounded soldiers and refugees in South Africa was only formulated at the end of last week, it has already assumed large dimensions. Knowing that the subject would be an interesting one to English nurses, I called at Walsingham House, Piccadilly, to ask the hon. secretary to furnish me with a few particulars.

Mrs. Blow had promised to see me at half past two, but three o'clock had struck before she could get away from the meetings in connexion with the movement. She admitted, as she came in hastily, that she had not had time for lunch, so whilst she went downstairs for the purpose, her husband kindly acted as spokesman.

"Mrs. Blow is, I expect, very much occupied?" I said.

"Indeed she is," was the rejoinder. "She has not a moment just now she can call her own, and Lady Randolph Churchill, the chairwoman, and Mrs. Ronalds, the treasurer, are equally hard pressed. Lady Randolph is taking a special interest in the project because her son is engaged in the war."

"When do you hope to get the ship started?"

"If all goes well in three weeks time, but of course there is a great deal to be done before then."

"The ship you have secured is called the 'Maine,' is it not?"

"Yes, it is a steamer of 4,200 tonnage, and is the twin vessel to the 'Missouri,' which was fitted out as a hospital ship in the Cuban war. The American Transport Line have behaved most generously. Not only have they offered the vessel to us free, but it is complete with officers and crew, and it is to be ours as long as the war lasts."

"It is certainly a princely offer. Do you anticipate any difficulty in collecting the £30,000 which will be required to equip the vessel?"

"Not the slightest, although no subscriptions are accepted except from Americans. Already large sums have been subscribed here and cabled from America, and the general feeling is one of satisfaction on the part of our countrymen and women that an opportunity has arisen by which we can show our sympathy with Great Britain, and our friendly feelings towards her."

"Where is the ship likely to be stationed?"

"Just wherever the War authorities think she can be of most use—Cape Town, Delagoa Bay, or Durban."

"Where is the 'Maine' at present?"

"Over here; and the plans for converting her into a hospital ship will probably be exactly the same as those prepared in the case of the 'Missouri,' only the work will be done in England. As soon as alterations are started the men will work day and night."

"Can you tell me about the doctors and nurses? I understand that the War Office urged that there were difficulties in allowing any but those of English nationality to occupy positions."

"At first this was believed to be so, but the authorities have been most kind in endeavouring to meet our wishes in every way. In all probability some will be English and others American. The matron is to be an English lady, but the four nurses under her will be from American hospitals. Only those whose training has been exceptionally complete will be accepted and we have cabled to Miss Clara Barton, whose position in the nursing world of America is well known, to make a selection for us of the most suitable candidates."

"Then, as yet, it is impossible to give me any names?"

"Quite impossible. There will be accommodation for 200 patients, but the nurses will be assisted by 40 non-commissioned officers and orderlies."

"I expect you will have offers of gifts in kind. Do you restrict these also to American firms only?"

"No, here we are glad any should help. With regard to drugs we have no anxiety. A well-known firm have volunteered to provide all we may require."

"Will the American refugees requiring medical assistance have the first claim to be taken on board the 'Maine'?"

"Decidedly not. The need for hospital nursing will be the only claim required. There is, unhappily, sure to be a good deal of sickness in the approaching summer, especially fever. On this subject Mrs. Blow has had some experience, as she lived three years in the Transvaal."

### Death in Our Ranks.

THE RISKS OF NURSING TYPHOID FEVER.

This week we have to announce with deep regret the death of three more nurses from typhoid fever.

At York the County Hospital and the Nurses' Home will greatly miss Nurse Marie Spraggon, who succumbed to typhoid contracted in the discharge of her duties at the sanatorium, Scarborough. Miss Spraggon had only recently completed her training at the County Hospital, where she had done extremely well. Her services were also much valued at the Nurses' Home, to which she belonged. In her earlier days Miss Spraggon had had the advantage of being brought into close contact with Louisa Lady Waterford, whose "Noble Life" so many nurses have read with interest. During the short time she did private nursing she gained the respect of all who worked with her. Her funeral was attended by a large number of her fellow nurses, and many loving tributes of flowers were laid on the coffin.

Nurse Mary Thomson, of the Sick Children's Hospital, Aberdeen, who had been in the institution five or six years, died from the same malady after an illness of a fortnight. The case was considered rather serious from the first. Miss Thomson, who was much esteemed, was only 24 years old.

The third death due to typhoid is that of Nurse Perrin. The recent appearance of the disease at Watton, in Norfolk, made it necessary to obtain the services of Miss Perrin, who was attached to the Fitzwilliam Street Home for Nurses, Cambridge. After successfully nursing her patient back to convalescence, she fell a victim to the disease, and died last Friday morning. A nurse who worked with her writes: "I may perhaps be allowed to say what a favourite she was. Her unselfish devotion to her work won universal respect. Nurse Perrin was an Irishwoman, and possessed all the 'sunny' characteristics traditional to that nation. Not a little did her brightness help to cheer the sick. In her hospital days Nurse Perrin was known among her friends as 'P. P.' (poor Perrin). Some years ago, when nursing a private case, she contracted diphtheria from her patient. So, of things in this world accounted unfortunate, Nurse Perrin had her full share. And yet she has died the death of a heroine, and more none of us could ask for or desire."

Nurse May Elsly died this week, after a short illness, at the Cottage Hospital, Iver, Bucks.

### Presentation.

The Hull Royal Infirmary.—Miss A. L. Cox, who leaves the Hull Royal Infirmary to take up her duties as lady superintendent at Monsall, has been presented with a travelling clock from the honorary staff, a silver tea caddy from the resident staff, a silver afternoon tea service, cake dish, caddy spoon and sugar tongs, a coalport hot-water jug, and bread and butter plate from the nursing staff past and present; and a silver serviette ring from the servants. Miss Cox's departure is much regretted by all, and she carries with her to Monsall every good wish for her future happiness and success.

### The Fevers of the South-West Coast of Africa.

By a Nurse.

I went out to the Congo in 1888, so I have seen and nursed a good many malarial fevers. You may perhaps like to know that my experience is that black-water fevers generally, and I think invariably, follow repeated attacks of ordinary malarial fever not properly treated, or not properly cured. In the early part of this year I heard someone remark that these fevers were caused by taking too much quinine. I made many inquiries of those who had had bilious hæmaturic fever or had nursed it, but in no case could I learn that more than a few grains of quinine had been taken before the fever began, and in some cases no quinine at all. One patient, however, when recovering, had a relapse of hæmaturia after every dose of quinine, and in this case eucalyptus was substituted with a good result.

On board the steamer on which I came to England a Swedish lady died from bilious hæmaturic fever, temperature 109·2 deg. Fahr. As I helped her friends to nurse her, I happen to know that the hæmaturia and high temperature began after a chill while she was in a feverish condition; it was the third hæmaturic fever in 18 months, and was fatal on the second day. The hæmaturia had almost ceased, but nothing could be got to touch the high temperature. This seems to have been the same with other fatal cases of which I have known. I wonder what is the general practice with regard to feeding patients with hæmaturia? I helped to nurse a lady just before leaving Congo, who had suffered from hæmaturia very severely, and she was fed from the first with milk, eggs, broth, &c. This is our usual practice, but I know that some doctors forbid almost all food until the fever can be subdued, and say that food only helps to keep up the temperature. There is one mission on the Congo where very little food is given at first; but there have been several deaths there from hæmaturia, and I cannot help thinking that the patient has a better chance of recovery if the strength be kept up from the first with nourishing food in liquid form.

My husband, an American Baptist missionary, who went to Congo in 1881, had a bad bilious hæmaturic fever after his first eleven months in Congo (at Banana), and as soon as he could be moved he was put on board to come to England. He has only twice had the slightest suspicion of a return of it, each time just before his furlough, and in consequence he hurried off a little more quickly. He can still do a much harder day's work than some who have been a much shorter time in the Tropics.

With regard to the mosquito theory, last December I went six hours' walk back from the river—150 miles above Stanley Pool—and slept in a tent two or three nights, walking to a different place each day, in a district where none of our party (some eighteen natives besides my husband and self) heard or saw a single mosquito; but one of our native boys knocked up with fever, and was not able to do anything for a week or so. I do not deny the presence of common flies in that district, but I do not remember that they were more troublesome than usual. I should like to know how to recognise the particularly obnoxious mosquito—I do not understand the meaning of its name. We have large and small black mosquitos, and large and small grey and striped mosquitos in Congo; but I don't know whether they all belong to different families. We have also a very minute fly (at first I took it to be a baby mosquito) which bites more severely than any mosquito I have ever seen, and the worst of it is that it gets through the finest mosquito netting. Nothing short of muslin will keep it out; it usually pays its visits at mid-day, when one wants to rest. About three years ago I visited a mission station, where fine young trees of Eucalyptus globulus were growing on three sides of the house. I was told that few mosquitos entered the house, and that all that came, visited the side where there were no eucalyptus trees. I noticed some time ago that Dr. Ross wanted to find an animal which was bitten by mosquitos. They bite goats badly, and I expect also antelopes and buffalo.

### Nursing in Fever Hospitals.

By an Ex-Matron of the Metropolitan Asylums Board.

I have followed with interest the controversy now going on about the nursing in the Metropolitan Asylums Board hospitals. Opinions have been freely expressed both from a charge and an assistant nurse's point of view, which are certainly conflicting. I quite agree with a "Trained Charge Nurse" that there must be something decidedly faulty about a system which cannot obtain order and concord between its several grades of nurses. I should be most sorry for the unfortunate matron who had to manage such a staff. May I ask if this is supposed to be the internal condition of the Board's hospitals? I think not, unless they have altered very considerably in a very short space of time.

In a large nursing staff one must occasionally come across unsuitable people in every rank of the nursing world who are quite unfitted for their posts. One often wonders why they have chosen the nursing profession, when they are so obviously unsuitable. This, unfortunately, is not confined to the Board's hospitals; they are to be met elsewhere in goodly numbers. A good deal also has been said and written about the difficulty of getting nurses and keeping them under the Board. I think most of the Board's matrons will agree with me, when I say that I never had the least trouble or difficulty in getting good charge nurses at any time from well-known training schools, and, in my humble opinion, they stayed as long as could be anticipated. One naturally expects to have more changes in a special than a general hospital. I think in those hospitals where the charge nurses are not expected to do night, they stay much longer than in those where alternate day and night duty is expected. It stands to reason that a charge nurse cannot take the same amount of interest in her ward when every three months she has to give place to another charge nurse, who perhaps works on entirely different lines. Of course, if good charge nurses who will take the trouble to train and teach their assistants and maintain good discipline in their wards cannot be obtained, the assistants cannot be expected to be satisfactory or to be a credit and comfort in the hospital. I think a good deal depends upon the sister or charge nurse, as the case may be. A bad sister or charge nurse will not turn out good nurses. She cannot do so unless she sets them a good example in all things, and is a good manager herself. I constantly see instances of a sister who has not got all the good work she might from a nurse or probationer, simply because she has not taken the trouble to manage her properly. If those in charge of wards would realise this, we should have a much better result than at present. I think much difficulty exists in getting first assistant nurses, and each year the difficulty increases. Very few general hospitals now train for less than three years; the Board asks, therefore, for what is not in the market under the present recognised training regulations of general hospitals. The second assistant nurses were always plentiful, and I found I could get suitable candidates by selecting them with discretion. The assistant nurses had classes and lectures during the winter months, and I found them, with few exceptions, most willing to learn all they could. At the end of their second year many of them were quite ready for promotion to the rank of first assistant nurse and were, as a rule, more satisfactory than those obtained elsewhere. Some, however, did not wish to stay on, and others went in for general training at the end of their second year. I think if the authorities could see their way to bind assistant nurses for a certain time (say three years), and would grant them a certificate for fever nursing at the end of that period, it would be a decided encouragement for second assistant nurses to remain on in the hospitals as first assistant nurses. They should be required to spend a certain time in each branch of fever work, and to pass a satisfactory examination after their course of lectures before the certificate is granted. I am of opinion that experience in fever work should form part of every nurse's training, more especially if a nurse is going to do private nursing. If arrangements could be made with general hospitals and training schools to give their probationers experience in fever nursing much good would result for the nursing profession. The Metropolitan Asylums Board hospitals are fine buildings, replete with every comfort and convenience for nurses as well as for patients, and I believe that they compare favourably with other hospitals, both as regards nursing and discipline.

### Examination Questions for Nurses.

### Rules.

THE competition is open to all. Answers must not exceed 500 words, and be written on one side of the paper only. The pseudonym, as well as the proper name and address, must be written on the *same* paper, and *not on a separate sheet*. Papers may be sent in for fifteen days only from the day of the publication of the question. Failure to comply with these rules will disqualify the candidate for competition. Prizes will be awarded for the two best answers. Papers to be sent to "The Editor," with "Examination" written in the left-hand corner of the envelope.

### Result of October Examination.

Nurse Gwytha is the winner of the first prize, and Nurse Molly of the second. The answers this month are good, showing that the candidates understand the subject on which they are writing, but, as usual, in almost all cases they are not explicit enough. It is well to remember that in teaching others, vague directions, easily understood by the initiated, fall flat on the ears of a probationer just entering the work, or on an amateur help, who may be an assistant in private work. I will give you an illustration of my meaning. Nurse Elizabeth would have gained the first prize, as her paper is very decidedly the best sent in, but unfortunately she entirely omits to mention how the fomentation is to be wrung out. Knowing the method so well she forgets that others are not always so well informed as herself, Therefore, good as her answer is, she fails. It is always a snare to professional nurses to forget that what is simple as A B C to them is Hebrew and Greek to the uninitiated. Again, Nurse Molly would have come out first instead of second but for an omission. Though her practice shows her knowledge of the necessity of putting the bandage under the patient's back before the fomentation is prepared she forgets to say so. Her answer is very good, and out of 147 papers hers is the only one that speaks of the advisability of letting the hot flannel sink gradually on to the patient's skin. Once more it is necessary to advise you all to be more careful in writing, spelling, and general tidiness. If two answers are equally good, but one is carefully sent in and the other carelessly, the examiner will naturally award the prize to the tidy candidate, for slovenliness in sending up examination papers augurs a general tendency to disorder in work of all kinds—a fatal thing in the nursing profession. Believe me, what is worth doing at all is worth doing well.

### First Prize.

Before applying a fomentation to the abdomen prepare the patient by adjusting a many-tailed bandage of flannel round the lower part of body, and have at hand a piece of protective (thin macintosh or jaconet) large enough to cover the fomentation and extend about an inch beyond all round, and safety pins for fastening the bandage. A small roller towel with a stick inserted in each end makes a good wringer. Place the centre of this in a large basin, on it the material for the fomentation, lightly folded, and pour straight on to it the boiling water, then twist the ends of the wringer in opposite directions till the water is squeezed out, take out the fomentation, shake it gently out, and place on the abdomen, covering it with the protective, finally fastening the bandage and replacing the nightdress (a flannel one preferably) and the bedclothes. The fomentation should be prepared, if possible, in the same room as the patient, and the kettle taken straight off the fire when first boiling, while all should be done as expeditiously as possible. The material used for a fomentation generally consists of coarse white flannel (in district work an old piece of blanket can often be obtained), even house-flannel will do, and gamgee tissue, spongio-piline, &c., are used. Flannel, unless very thick, may be doubled when applied; red or coloured should not be used. Poppy-head fomentations have the poppy heads (six to a pint of water) boiled in the water, and turpentine, laudanum, &c., may be mixed with the water, or sprinkled quickly on the surface of the fomentation. Fomentations generally require renewing pretty often, and a second one should be wrung out ready to apply, before removing the first, though the bandage should be unfastened before this is prepared. The first one and wringer should then be thoroughly dried for future use, but the bandage should not need changing.

### Second Prize.

1. Two or three thicknesses of coarse flannel or blanket, a piece of mackintosh, cotton wool, a wide flannel binder, a wringer or towel, and boiling water.

2. Place a towel or wringer over a basin, and in it the flannel folded to the required size. Pour over a quantity of boiling water, take hold of the two dry ends of the towel and wring until the flannel is as dry as possible. Before putting on the patient, shake the flannel so that air may get between the folds, which prevents scalding; if then too hot, hold it slightly raised off the skin for a minute, then let it down gently and cover with mackintosh a size larger than the flannel; over that put a warm piece of cotton wool, secure all with a flannel binder, firmly pinned round. When turpentine or laudanum is ordered, the quantity should be quickly sprinkled on the flannel before applying to the skin. Spongio-piline is sometimes used instead of flannel for fomentations, being wrung out in the same way, but does not require covering with mackintosh, as one side of it is waterproof.

### Questions for November.

1. Give an account of what you would consider the most satisfactory room in which to nurse a patient with a protracted illness.

2. What do you consider the most suitable position for the sick room of a patient suffering from infectious disease?

N.B.—These questions were so indifferently answered in the spring that no prize was given. It is desirable that the same candidates should send up papers again.

### Where to Go.

THE EMPRESS ROOMS, ROYAL PALACE HOTEL, KENSINGTON, Tuesday, November 28.—The annual ball, in aid of the funds of the Metropolitan Ear, Nose, and Throat Hospital. Tickets, 10s. 6d. each, from the secretary.

### Echoes from the Outside World.

AN OPEN LETTER TO A HOSPITAL NURSE.

The war naturally remains the subject uppermost in the minds of English women to-day, and many a heart, I fear, is much heavier this week than it was when last I wrote. The account of the losses at Elandslaagte and Rietfontein have been published, and though the officers have not suffered so severely as at Glencoe, the list of killed and wounded is a lengthy and a sad one. The intelligence of Sir William Symons' death, after he had lived long enough to afford ground for hope, seemed to make us more sorrowful—both for the sake of Lady Symons and for that of the army —than if he had died at once, though one cannot but be glad that he was able to know of his promotion, and how the people of England thanked him for all he had done. On Tuesday the news of the battle at Ladysmith came, and with it the startling details of the capture by the enemy of the Royal Irish Fusiliers, the Gloucester Regiment, and No. 10 Mountain Battery after terrific fighting.

Englishmen know how to meet a reverse; and though the reverse which we undoubtedly sustained in the encounter required the exercise of some of the highest national qualities, most people seem to have risen to the occasion. I was, however, very sorry to see that some few attacked General White, in spite of his manly letter, even before the despatch, which put a different complexion on the matter, was received. It is said that "somebody blundered." Possibly. So did someone at the charge of the Light Brigade at Balaclava. But that did not hinder the war from being brought to a successful issue; and all we can do is to pray that the reinforcements may arrive early enough to prevent our troops from having to fight a second time against such tremendous odds. At all events, we have the consolation of knowing that Sir Redvers Buller, whose reception at Cape Town seems to have been simply magnificent, is now on South African soil.

I am afraid there is not the remotest chance of our men being back in December, though General Yule's mother, a dear old lady of over eighty years of age, still hopes that her son will be home for Christmas. Some one who saw her at her house in Ealing, where she has lived for fifty years, describes her as just what an English soldier's mother should be. Cheerful and bright, though not quite able to hide her anxiety, and trying to drown her trouble by a feeling of pride that her son should be fighting for his Queen and his country. At a time like this it is wonderful how the touch of nature makes us all kin. A pretty little story is told of Lady Dudley at the War Office. She had gone to scan the list of dead and wounded, and noticed a poor feeble old man trying in vain to obtain a glimpse of the board. Every time he attempted to get near, she saw him pushed away again, and going up to him she asked if she could be of any help. Ascertaining that he was anxious about his son, she promised to bring him the news herself, if he would go home. She must almost have regretted her suggestion when she read the name of the young fellow amongst the dead; but she kept her promise, and drove a considerable distance to break to the poor father the loss he had sustained.

The visit of the German Emperor, who arrives at Windsor on the 20th, is inevitably associated with the situation in South Africa. There is, of course, no reason why he should not be the guest of his grandmother at any time, but it is pretty clear that the present has been selected for reasons not altogether of a family and personal character. Moreover, the visit is to be paid in spite of the opposition of a section of the German Press, and is not to be confined to a stay of a few days at the Palace. The Kaiser is to be seen in London, at Oxford, and possibly in the North of England. There is every reason to hope and believe that wherever he goes while he is among us he will meet with the very cordial reception which his friendliness towards England under not very easy conditions merits.

Except in India—where the Viceroy, Lord Curzon, has been doing his best to take a peep behind the scenes by paying surprise visits to the various relief works in the neighbourhood of Delhi—the plague appears to be dying out. A private letter from Hongkong says that there is hardly a single case left, and Lord Cromer telegraphed last week that it is three weeks since anyone has fallen a victim to plague in Alexandria. Evidently the measures taken to prevent the malady spreading have been carefully carried out, for it has practically never gone beyond Alexandria. The authorities will now reap the reward of their vigilance, for instead of Egypt being deserted, those whose health prevents them wintering in England will probably flock in as large numbers as usual to Cairo and the Nile.

Once more the dogs are parading the streets of the metropolis with unmuzzled countenances. As yet they can hardly grasp the situation, and must believe in their canine hearts that their owners have forgotten this erstwhile necessary article of their outdoor attire. I hope sincerely that the dogs are content, because I have come across several ladies who, though they originally inveighed loud and long against the Muzzling Order, are crying out yet more loudly since it has been revoked. They complain that, whereas last week they could walk along without anxiety, knowing that their pets could not be set upon by bigger dogs, nor could they, in their turn, show their spirit by biting children's legs, now the daily walk has become a time of fear. As if to revenge themselves for the long period during which their freedom was curtailed, those dogs naturally savage appear to have grown much fiercer, and even the peaceable animals have celebrated the occasion by lawlessness and unruly behaviour. It was only on Friday that the Muzzling Order was rescinded; on Saturday four persons were treated at St. Thomas's Hospital alone for bad dog bites. Three of these victims were women who had been proceeding quietly along the streets, and had been attacked apparently for no reason except that the dogs were pleased to have regained the power of using their teeth. I should not be surprised if, in the course of a few weeks, the President of the Board of Agriculture is asked to receive a deputation from harassed ladies wishful to re-impose the muzzle.

Many of us have, at some time, spent a pleasant hour or two with Florence Marryat. Her books are just the kind to take up when one is too tired to concentrate attention on anything which requires the exercise of the brain. Mrs. Francis Lean—the deceased author married Mr. Lean as her second husband—always wrote pleasantly and interestingly, and an arm-chair, a nice fire, a little leisure, and a book by "Florence Marryat" secured for many a tired worker a treat on a winter's afternoon or evening. Had we realised what a busy woman the writer was, I think we should have been doubly interested in the famous Captain Marryat's clever daughter. Married at sixteen to Captain Ross Church, she wrote her first novel, "Love's Conflict," in her spare moments, when nursing her children through scarlet fever, as a distraction from anxiety. As her first attempt proved successful, it was followed by others in rapid succession. How she managed to turn out so many volumes must be a matter of surprise to those who can themselves only work slowly, for her household duties with eight children could have been no sinecure. In addition to these, as well as writing novels, she composed plays, and sometimes acted in them herself; she wrote for magazines and newspapers; and three times toured the country with an entertainment of her own arranging, besides paying a visit to America, where she was much appreciated.

### A Concert by Incurable Children.

An Edinburgh correspondent sends us an interesting account of a concert given by the children in the "Nursery" of the concert given by the children in the "Nursery" of the Edinburgh Incurable Hospital. The "Nursery" is the children's ward. Our correspondent says:—"A friend of one of the medical officers took great trouble to teach the little ones songs and recitations, and helped them to get up two charades. They had an unlimited supply of art muslin, bought with their own pennies, which they had made up into little evening frocks; but the gaily-coloured attire only made their white faces look all the paler and more pathetic. Do you wonder that I had difficulty in restraining a tear as those poor children sang in a chorus, 'We will not join the dance'? Dear little squint-eyed Peggy, minus a leg and arm; Jack, with no foot, and only one knee; few if any of them with their limbs entire, and these otherwise disabled. Still they looked so happy that I have no doubt they all would have tried, at least, to join in any dance—wooden legs, crutches, stumps, and all! It was a great pleasure to the children seeing all their doctors among the audience, and to have for their chairman the surgeon who had so carefully and so beautifully removed their little limbs, which had been but painful encumbrances to them before. In one of the charades, "Longmore," they enacted the scene they are perhaps more familiar with than any other in this life, namely, nursing the dying. The relative positions of the doctor, staff nurse, probationer, and patient were amusingly pourtrayed. In another charade Jack and Elsie were crowned King and Queen. They had only one leg between them. Perhaps the funniest thing of all was when three of them sang a fairy song. Their joy was complete when they were asked to have tea in the matron's room, with their nurses and the maids waiting upon them, 'Just like real ladies, you know!' Though the entertainment was amusing and bright it was strongly tinged with sadness."

### Appointments.

Eastern Fever Hospital, Homerton.—Mrs. E. M. Day has been appointed Matron. She was trained at Lincoln County Hospital. Subsequently she served as night superintendent and ward sister at the Metropolitan Hospital, Kingsland Road; housekeeper at the South-Western Fever Hospital, Stockwell; and matron of Gore Farm Convalescent Fever Hospital, Darenth.

Basingstoke Cottage Hospital.—Miss M. Baker has been appointed Matron. She was trained at the Royal Hants County Hospital, Winchester, and has since worked on the staff.

### Minor Appointments.

City Isolation Hospital, Nottingham.—The secretary, in sending us details of the previous appointments of Miss Netta Allan, the new Sister, stated that she had acted as "temporary matron and housekeeper" at Victoria Park Hospital. This, Miss Allan explains, was a mistake; she acted as "temporary assistant matron and housekeeper."

Guildford Workhouse Infirmary.—Miss Edith Hughes, who has been appointed Superintendent Nurse, wishes us to add to the information given last week that at the end of her training at Mill Road Infirmary, Liverpool, she was appointed charge nurse of a female medical and the maternity ward, which post she held for three years and a-half. She has had experience in private nursing, and holds the L.O.S. certificate.

### For Reading to the Sick.

ALL SAINTS' DAY, NOVEMBER 1st.

"What are these which are arrayed in white robes—and whence came they?

"These are they which came out of great tribulation, and have washed their robes and made them white in the blood of the Lamb. Therefore they are set before Him day and night in His temple; and He that sitteth on the throne shall dwell among them. . . . They shall hunger no more, neither thirst any more; and God shall wipe away all tears from their eyes.—*Rev.* vii. 13, 14, 16, 17.

The Saints of God! their conflict past,
And life's long battle won and lost,
No more they need the shield or sword,
They cast them down before their Lord:
O happy Saints! for ever blest,
At Jesu's feet how safe you rest.

The Saints of God! life's voyage o'er,
Safe landed on that blissful shore,
No stormy tempests now they dread,
No roaring billows lift their head:
O happy Saints! for ever blest,
In that calm haven of your rest. —*Maclagan.*

**Reading.**

As there is a perfect union betwixt the glorious saints in Heaven, and an union, though imperfect, betwixt the saints on earth, so there is an union, partly perfect and partly imperfect, between the saints in Heaven and the saints below upon earth; perfect in respect of those glorified saints above, imperfect in respect of the weak returns we are able to make to them again.

Let no man think that, because those blessed souls are out of sight, far distant in another world, and we are here toiling in a vale of tears, we have therefore lost all mutual regard to each other; no, there is still, and ever will be, a secret but unfailing correspondence between Heaven and earth. The present happiness of those heavenly citizens cannot have abated aught of their knowledge and charity; but must needs have raised them to a higher pitch of both. They, therefore, who are now glorious comprehensors cannot but, in a generality, retain the notice of the sad condition of us poor travellers here below, panting towards our rest, together with them; and, in common, wish for the happy consummation of this our weary pilgrimage, in the fruition of their glory. That they have any perspective, whereby they can see down into our particular wants, is that which we find no ground to believe: it is enough that they have an universal apprehension of the estate of Christ's warfaring Church upon the face of the earth;* and fellow-members of the same mystical body long for a perfect glorification of the whole.—*Bishop Hall.*

I think of those saints I have known, and lift up mine eyes
To the far-away home of beautiful Paradise,
Where the song of saints gives voice to an undividing sea,
On whose plain their feet stand firm while they keep their Jubilee.
As the sound of waters their voice, as the sound of thunderings,
While they all at once rejoice, while all sing and while each one sings;
Where more saints flock in, and more, and yet more and again yet more,
And not one turns back to depart thro' the open entrance-door.
O sights of our lovely earth, and sound of our earthly sea,
Speak to me of Paradise, of all blessed saints to me;
Or keep silence touching them and speak to my heart alone,
Of the Saint of Saints, the King of Kings, the Lamb on the Throne. —*Christina Rossetti.*

* Rev. vi. 10.

### Everybody's Opinion.

**[Correspondence on all subjects is invited, but we cannot in any way be responsible for the opinions expressed by our correspondents. No communication can be entertained if the name and address of the correspondent is not given, as a guarantee of good faith but not necessarily for publication, or unless one side of the paper only is written on.]**

### BABY'S CORD.

"PERPLEXED" writes: Will you please let me thank all the nurses who so kindly sent the result of their experience in dressing baby's cord. I am daily expecting a call to another case, and shall this time try the daily dressing and powdering. My great ambition is to arrive at perfection or as near that as possible in my treatment of the mother and child, and I feel sure that all those who took interest in my perplexity will be equally glad to hear that I have achieved my purpose when the case is over and I am at liberty to write again. I also thank you for giving me space.

"NURSE AGNES" writes: I think that "Perplexed" will find the following method of dressing the cord very satisfactory. Cut a square piece of old linen about five inches each way; make a hole in the centre and pass the cord through it; fold the linen neatly over the cord and turn it up. Put a small pad of absorbent wool under the linen next the abdomen to prevent the hard cord hurting the infant, and another pad of wool over the linen to press the navel flat. The cord should be well dusted before wrapping in the linen with zinc and starch powder, which dries up all moisture much better than boracic. The cord should be well dusted, and the piece of linen changed, every morning. This keeps the navel dry and sweet, and when the cord comes off between the fourth and seventh day the navel is perfectly flat and healed. I usually put a pad of wool over the navel before putting on the binder for about a fortnight after the cord comes off, to keep it flat. I was trained in two of the largest lying-in hospitals in London, and have had twelve years' constant experience in maternity work only, and for the last ten years, since using the above method, have never had the least trouble with the baby's cord.

### TRAINING IN SMALL HOSPITALS.

"BUSY BEE" writes: Being a regular reader of your valuable paper, and also of the various grievances of various nurses and the British public's treatment of them, I beg to air a grievance of my own. I notice that one of your correspondents mentions training in small hospitals. I myself was trained in a small hospital with about 50 beds; since then I have done a good deal of district work in the East End of London (which in itself is very good training, as any one who has worked there can testify), and have also worked in private nursing with nurses from most of the London hospitals. But when I applied to the Nurses' Co-operation for a post, because they wanted thirty nurses to go to Johannesburg, I was told that they had advertised for fully-trained nurses. It is very galling when one has done hospital and private nursing for eleven years that this should count for nothing.

### WORKHOUSE NURSING.

"E. R. W." writes: I fear that I must still maintain my attitude, even at the risk of appearing unkind. Covering a wound does not heal it. It remains a fact that, apart from the large infirmaries, workhouse nursing is still in the hands of the half-trained and the uneducated, and I maintain again that as long as this is so progress in workhouse infirmaries will be, if not stationary, limited. My letter is an appeal for a larger number of ladies to enter the work. The master and the matron are constantly made the objects of attack, but in the cases I have had an insight into they have been vastly the superiors of the nurses and the superintendent, which to my mind is a great pity, for it cripples the work. Truth is always wounding, and if I have said anything to hurt anyone's feelings it is because the workhouse infirmary patients are nearest to my heart. I do not consider that "professionally" (in the sense I meant it) army nursing stands even as high as infirmary nursing.

### ONE OF THE FAMILY.

"R. A. G." writes: Being a constant reader of your columns I should like you to insert my opinion on this subject. I am a nurse of considerable experience, having taken private cases in the north, midlands, and south, and in almost every instance I have been treated with as much consideration as the case would permit. As to being "one of the family," in some houses, as "Beth" stated, it is preferable not to be treated so—for instance, a rough-and-ready cottage home in Yorkshire, where all the family helped themselves with their own knives and forks out of the dish. Had I have been at the table it would have been extremely awkward. Then again, you meet folk who wish you to mix with them to save a little extra trouble, and all the time do not make you feel at home; this type would please me better by allowing me to eat and sit alone. Or another instance: A woman said to me once, "There is a bath for the servants upstairs," to which I made answer, "That is very nice; for it is not always convenient for them to use the one we use." It is far pleasanter not to associate much with the class of persons who do not understand the position of a nurse in the house. I am glad to say that in my experience most of the friends of my patients have been of the more genial type. As a rule, I endeavour to give as little extra work and suit myself to the arrangements of the house as much as possible, and in cases of necessity doing things which do not come under the heading of nursing. "Vera" forgets that although the nurse is a stranger in the house, to her often is entrusted the life of the bread-winner of the family, or, if not the father, a very dear member. The nurse must be diligent in her duty for often 14 or 15 hours at a time without relief, except for the necessary time for meals. I think if "Vera" changed places with nurse she would be glad of a little time amidst other surroundings and other faces. May I add that people to whom a nurse is such a bugbear as "Vera" represents would be far happier if they nursed their sick themselves.

### THE TRAINING IN WORKHOUSE INFIRMARIES.

"A NURSE IN ONE OF THE HOSPITALS UNDER THE METROPOLITAN ASYLUMS BOARD" writes: A good deal of attention has been given lately to the training in workhouse infirmaries. I received my certificate from one of our large infirmaries, and should like to tell you a little about it. Our matron is well known in the nursing world, our sisters were drawn from both London and provincial hospitals, and they spared no pains to make their nurses excel in practical work. As they had more than one ward under them much of the management fell to the staff nurses, and thus most valuable experience was gained by them. Lectures were given by the matron and doctors, not only on nursing, physiology, and anatomy, but we had the advantage of many a clinical lecture, when symptoms of disease, treatment, and the use of drugs were taught. As there are no students at infirmaries (and I have heard them bemoan the fact) all practical work is done by the nurses from urine testing to helping at operations. Then the final examination. I rather fancy many a hospital nurse would come off badly, for theory will not carry you through. You must pass a stiff examination in practical work or the coveted certificate will be lost. Besides all this there are many great advantages; you nurse your patient to the very end of his illness, and there is no turning out for want of room which is so unavoidable in hospital. Then the much-despised chronic cases. Of what is this class largely composed? All forms of paralysis, lung and heart disease, gout, and even muscular atrophy and inco-ordination, many of which are never seen inside a hospital. From a purely nursing point of view they are not to be despised, for they require the best of care, and often it is no easy matter to tempt the capricious appetites of chronic cases. Or look at another side of the question. Those who take up nursing from love of the suffering ones can find an endless scope for their energies, for it is the hopeless and helpless who provoke your pity, patience, and tenderness; but one is amply repaid when one can ease their pain in the least degree. Certainly there is nothing to look down on in the training. I have worked in large and important hospitals since those days, but I have never once regretted that I trained in a workhouse infirmary.

### Notes and Queries.

**The contents of the Editor's Letter-box have now reached such unwieldy proportions that it has become necessary to establish a hard and fast rule regarding Answers to Correspondents. In future, all questions requiring replies will continue to be answered in this column without any fee. If an answer is required by letter, a fee of half-a-crown must be enclosed with the note containing the enquiry. We are always pleased to help our numerous correspondents to the fullest extent, and we can trust them to sympathise in the overwhelming amount of writing which makes the new rules a necessity.**

**Every communication must be accompanied by the writer's name and address, otherwise it will receive no attention.**

*Visiting Nurse.*

(49) Would you kindly inform me where I can obtain information regarding the appointment of trained nurses as visiting inspectors of the children attending the London Board schools?—*School Board.*

The Hon. Secretary, The London School Nurses' Society, Ivy Hall Richmond, S.W., would be able to give you full information.

*Midwifery.*

(50) Will you kindly inform me where I could attend lectures and demonstrations in midwifery? I have little time available before six p.m. If you could recommend me a good book I should be greatly obliged. —*E. T. T.*

The Midwives' Institute, 12, Buckingham Street, Strand, have constantly courses of lectures on the subject. 2. You will find "A Practical Handbook of Midwifery," by Haultain (Scientific Press, price 6s.), one of the best.

*Dandruff and Constipation.*

(51) Emily J. kindly sends the result of her experience with dandruff, trusting that it may be useful to our querist of some little time ago. After recovery from a long illness, the patient—an old lady of 63—seemed in danger of losing all her hair. Miss J. divided the hair and rubbed the scalp with the freshly-cut sections of a lemon, and afterwards brushed the hair free from the bits of pulp left in. The hair was thoroughly dried before being put up. This treatment was pursued daily until the hair became longer and thicker than it had ever been before. This correspondent also adds that she has found in her own case that the yolk of an egg well beaten and mixed with half a pint of milk heated up to boiling point only, a remedy for habitual constipation. She takes this regularly, and finds it as effective as the daily dose of cascara to which she had been accustomed for two and a half years.

*Sloyd.*

(52) Can you kindly tell me the meaning of the word "Sloyd"? I believe it is a term either in reference to education or medicine.

Sloyd is the name of a system of teaching handicrafts. It is one of the Swedish methods of hand and eye education, and lately a number of English ladies have made it an object of study. Consult a good encyclopædia for further information.

*Pamphlets.*

(53) 1. Will you kindly tell me if there are any cheap pamphlets on the desirability of vaccination? I am a midwife and always advise my patients to have their babies vaccinated, but generally meet with some objection; I think if I could give them some easy, sensibly-written paper about it they might be persuaded to have it done. It is generally the fathers who object. 2. Will you also tell me if children are vaccinated now from other children, as this seems to be the chief cause of objection?—*C. C. P.*

The Jenner Society, Gloucester, has been founded "To collect, diffuse, and popularise knowledge in regard to the history of smallpox and the value of vaccination as a protection against it; to promote the practice of vaccination in a safe and efficient manner; and to further generally the adoption of those modes of preventing and treating disease which rest upon the foundation of Jenner's great inquiry." The little booklet, "The Cry of the Children," published by it is very taking and convincing; but you may have a sample of the publications by applying to the society and select those most suitable for your purpose. 2. No; children are now vaccinated by the public vaccinator with calf lymph specially prepared under Government inspection.

*M. A. B.*

(54) Could you tell me which of the "Metropolitan Asylums Board Fever Hospitals" engage permanent day "charges"?—*Nurse.*

The Metropolitan Asylums Board, Norfolk Street, Strand, are constantly advertising for charge nurses for their various hospital and asylums. Write direct to the secretary or consult our advertisement columns.

*Surgical Training.*

(55) Would you kindly oblige me with some information as to where, or in what hospital, I could get a year's surgical training, free, if possible, but I am willing to pay a premium?—*E. C. D.*

Have you read our advertisement columns? There are several special hospitals which grant certificates for either one or two years where you could obtain the training free, or even be paid a salary. Consult "The Nursing Profession: How and Where to Train." Select the hospital most likely to meet your requirements, and apply in the usual way. The book is published at 2s. by the Scientific Press.

(56) Can you tell me at what hospital I can get entirely surgical training, as I want to be only a surgical nurse? Is it possible to get such a training and certificate, and at what London hospital?—*E. S.*

A purely surgical training does not make a "trained nurse." The Poplar Hospital for Accidents, Poplar, E., would be about the best for you. The certificates are for two years.

*Grants to Nursing Associations.*

(57) My nursing association are applying for a grant from the Board of Guardians, and we are told that the application would be considered more favourably if precedents could be quoted. Could you let me know of instances in which such help is given, and also what amounts are granted?—*Hon. Treasurer.*

The Local Government Board frequently sanctions grants by the Guardians to nursing associations. Apply for particulars to the Secretary, Whitehall, S.W.

*Rash.*

(58) Will you kindly tell me (1) if persistent use of acetanilide, phenazone, or phenacetin in a case of enteric fever would produce a rash resembling scarlatina? 2. Also, does potassium bromide in repeated doses produce a rash over the body?—*A. P.*

The use of coal tar antipyretics such as those mentioned sometimes causes a rash, but it must not be supposed that a roseola occurring in the course of typhoid fever is of necessity the result of the drugs taken, as various rashes are apt to occur during the progress of that disease, possibly as the result of toxins absorbed by the intestines.

*Weak Intellect.*

(59) Could you tell me of any institution or home where a girl of 22, who has a weak intellect and a cleft palate, could be received permanently? She is able to work for her living under supervision, and is quite respectable, but is not sufficiently bright to go out to service and make her own way in the world. She is an orphan.

Write to the National Association for Promoting the Welfare of the Feeble-minded, 49, Victoria Street, S.W., for advice, and possibly help.

*Invalid Cookery.*

(60) I shall be much obliged if you will let me know in your "Notes and Queries" column of a good book on invalid cookery; also of a dictionary (small edition) of medical terms.—*H. M. S.*

"The Art of Feeding the Invalid" (Scientific Press, price 3s. 6d.) might suit you for the former; and Miss Honnor Morten's "The Nurse's Dictionary of Medical Terms" (same publishers, 2s.) for the latter.

*Young Probationers.*

(61) Will you kindly oblige by informing me if you can recommend any training school or institution for preparatory work for intending nursing probationers between 19 and 20 years of age?—*Pater.*

It is usual for young probationers to try and enter a children's hospital, as the only preparatory training schools are in connection with our largest hospitals, and for the benefit of their own probationers. You will find full particulars in "The Nursing Profession: How and Where to Train" (the Scientific Press), price 2s.

*Maternity Training.*

*Alpha.*—In addition to the lying-in hospitals mentioned last week, the City of London Lying-in Hospital, City Road, has 36 beds.

### TRAVEL NOTES AND QUERIES.

**Rules in Regard to Correspondence for this Section.—All questioners must use a pseudonym for publication, but the communication must also bear the writer's own name and address as well, which will be regarded as confidential. All such communications to be addressed "Travel Editor, 'Nursing Mirror,' 28, Southampton Street, Strand." No charge will be made for inserting and answering questions in the inquiry column, and all will be answered in rotation as space permits. If an answer by letter is required, a stamped and addressed envelope must be enclosed, together with 2s. 6d., which fee will be devoted to the objects of the "Hospital Convalescent Fund." Any inquiries reaching the office after Monday cannot be answered in "The Mirror," of the current week.**

Oberammergau (Stevenage).—I am now able to give you some information with regard to the Passion Play, but all arrangements are by no means completed; in the course of another six weeks Messrs. Gaze and Cook will give me particulars (not yet arranged) of their own conducted tours. Dr. Lunn's parties, as to which you inquire, start every week from May 8th until September 25th. Cost, £25 4s., for twenty days. A conductor accompanies the party out, but they return independently. Should anyone wish to return immediately after the Play on the twelfth day a reduction of £3 8s. will be made, which is the cost of the eight days' hotel coupons, less 10 per cent. Unless you are pressed for time I do not consider that this would be an advantageous arrangement, for you would have to provide your own hotel accommodation on the return journey. If you like to send me a stamped and addressed envelope I will forward you Dr. Lunn's account of the projected journey. See further answer to "Miracle Play."

Oberammergau ("Miracle Play").—You will see full answer concerning conducted tour in answer to "Stevenage." If you think of going independently, doubtless more agreeable but much more expensive, the return ticket, first class, to Munich is £7 16s. 3d.; second, £5 9s. 11d. From there trains run to Murnau, some 40 miles further (you can estimate the expense roughly, something like 15s. first return and 10s. second). From Murnau there is an electric tram to Oberammergau, or carriages from Oberau, the station beyond Murnau. The accommodation is somewhat expensive if you do not belong to a party, ranging from 10 to 15 marks. Tickets for the Play from 3s. to 10s., the same as marks. Later on I shall be able to give you still fuller information. Should you wish to go independently, there are those who make a business of booking seats for visitors. I can give you their addresses should you wish, if you send me a stamped and addressed envelope.

Ireland (Peony).—No, I fear I cannot encourage you. Travelling and hotel accommodation is dear. No doubt it might be managed cheaply by seeking quarters in the cottages, but I really think it would be impossible for ladies; you have no idea of the discomforts you would have to encounter. If you think you could face it, and tell me more of the locality you wish to visit, I will see what can be done.

